# Dendrite and Axon Specific Geometrical Transformation in Neurite Development

**DOI:** 10.3389/fncom.2015.00156

**Published:** 2016-01-28

**Authors:** Vasily I. Mironov, Alexey V. Semyanov, Victor B. Kazantsev

**Affiliations:** ^1^Department of Neurotechnologies, Institute of Biology and Biomedicine, Lobachevsky State University of Nizhny NovgorodNizhny Novgorod, Russia; ^2^Laboratory of Nonlinear Dynamics of Living Systems, Institute of Applied Physics of the Russian Academy of ScienceNizhny Novgorod, Russia

**Keywords:** shape specific elongation, neurite outgrowth, tubulin, active transport, microtubule cytoskeleton

## Abstract

We propose a model of neurite growth to explain the differences in dendrite and axon specific neurite development. The model implements basic molecular kinetics, e.g., building protein synthesis and transport to the growth cone, and includes explicit dependence of the building kinetics on the geometry of the neurite. The basic assumption was that the radius of the neurite decreases with length. We found that the neurite dynamics crucially depended on the relationship between the rate of active transport and the rate of morphological changes. If these rates were in the balance, then the neurite displayed axon specific development with a constant elongation speed. For dendrite specific growth, the maximal length was rapidly saturated by degradation of building protein structures or limited by proximal part expansion reaching the characteristic cell size.

## Introduction

Recent theoretical and experimental studies in neuroscience have demonstrated that many molecular, cellular, network, and system level functions of the brain can be simulated using computational models *in silico*. Such models can assist in the understanding of how lower level dynamics can be projected to a system level outcome. These models involve construction of large scale networks mimicking the morphology and functions of particular brain circuits, such as models of thalamocortical system (Izhikevich and Edelman, 2008), cortical column simulation (Markram, 2006), and many other brain modeling initiatives (Fleischer et al., 2007; Ananthanarayanan et al., 2009; Markram et al., 2011). The models used in these simulations collected cell morphology, molecular interactions and connectivity from experimental studies. Some aspects of network development *in silico* can be investigated using simulators such as Netmorph (Koene et al., 2009) and CX3D (Zubler and Douglas, 2009; Zubler et al., 2013) and neural culture models (Gritsun et al., 2012). However, many fundamental questions, including axon, and dendrite differentiation and the influence of intra- and extra-cellular factors on the elongation, navigation, and branching of neurites, are still under discussion. From a computational point of view, it is also important to generate a minimal biologically relevant cell development model to simulate large networks.

Neurite growth involves complex molecular machinery responsible for cytoskeleton formation driven by intracellular and extracellular signaling. One of the main building blocks of the cytoskeleton are microtubules (Heidemann, 1996). They represent long polymers formed by heterodimers of tubulin. The most active microtubules, which are capable of joining and contracting, are localized in the end of the neurite called the growth cone (Shea, 1999; Morrison et al., 2002). Thus, neurite elongation can be considered as elongation of the microtubules located in the distant parts of the neurite. Building proteins (e.g., tubulin) are synthesized in the soma and have to be transported to the growth cone. In one hypothesis, this transport is provided by oligomers or microtubule fragments transported by motor proteins along the neurite (Wang and Brown, 2002; Vale, 2003; Baas and Buster, 2004; Hirokawa and Takemura, 2007). Another hypothesis is based on heterodimers of tubulin synthesized in the soma and moved along the neurite due to both diffusion and active transport (Galbraith et al., 1999; Kimura et al., 2005).

Different aspects of neurite growth dynamics have been studied using mathematical models. Most of them are focused on the description of molecular machinery underlying the elongation process. Such models usually consider tubulin microtubule dynamics and its contribution in neurite growth. All models describing the elongation dynamics, share the concept mentioned above (tubulin synthesis takes place in the cell body and then it travels toward the growth cone). The key difference of the existing models is in description of building protein delivery to the growth cone. In (Samuels et al., 1996; Van Ooyen et al., 2001; Toriyama et al., 2010) neurite is considered as a single compartment, and elongation dynamics is determined by a fraction of a some shared resource (such as building proteins or proteins promoting elongation). The competition process leads to the monopolization of the resource by single neurite which demonstrates axon-like development, while other neurites elongation is slowing down.

Another approach has been proposed in Miller and Samuels (1997). Based on conveyor analogy of active transport process, accounting the degradation of the protein, linear decrease active transport velocity has been proposed. Using this assumption, the estimation of the axon maximum length was performed.

More detailed description of the outgrowth development involves consideration of the evolution of the building proteins concentration profile along the neurite. In one approach the outgrowth is represented as a segments sequence and tubulin transition toward the growth cone is carried out by the adjacent compartments interaction (active transport and/or diffusion). It should be noted that usually the segments radius changes when the neurite branching occurs. These models reproduce a wide variety of developmental scenarios, for example dendritic trees formation accounting the calcium-dependent processes of MAPs (de-)phosphorylation (Graham and Van Ooyen, 2001, 2004; Hely et al., 2001; Kiddie et al., 2004; Hjorth et al., 2014; Mironov et al., 2014).

Another models type suggests the most detailed study of the tubulin transport along non-branching process. To this end, the PDE describing the concentration of tubulin as a function of time and distance from the soma along the axon to the growth cone is used (McLean et al., 2004; Diehl et al., 2014). The analysis of PDE provides the stationary tubulin distribution along the process and allows making estimations of neurite maximum length.

Here, a novel mathematical model reproducing experimental data of dendrite and axon specific development is proposed. The main focus is made on the relationship between geometrical characteristics and effective rate of tubulin transport of the neurite. In particular, the balance of these rates predicts a neurite projected over a very long distance, i.e., reproducing the axon specificity.

## Results

### Geometry independent model

We propose a mathematical model of non-branching neurite elongation based on the dynamics of cytoskeleton microtubules under the following assumptions. The elongation depends on tubulin concentration in the growth cone. Building proteins are synthesized only in the cell soma, and there is no additional tubulin production occur in other compartments (e.g., in the axon and dendrites). We assumed that the intracellular machinery regulating the synthesis compensates for building proteins utilized for the elongation (moreover, as we shall see, together with geometrical changes it forms a positive feedback that supports the neurite growth). In other words, the concentration of tubulin heterodimers in the soma is sustained near a constant level (Cleveland et al., 1981; Theodorakis and Cleveland, 1992). In the cell soma, the tubulin forms quite massive structures (e.g., oligomers or microtubule fragments), hence the contribution of their passive diffusion to the neurite elongation is negligibly small. Therefore, we assumed that tubulin transport from the cell soma to the growth cone was provided by active transport only. It is implemented by motor proteins binding tubulin structures and bringing them to the growth cone. For our purposes, we do not explore further details of these mechanisms, assuming only that the active transport occurs with a certain constant speed.

Also, it should be mentioned that the neurite elongation rate (Keenan et al., 2006; Rizzo et al., 2013) is usually several times lower than the active transport velocity (Table 1) and it makes possible to consider the growth process using quasi-steady-state approximation (i.e., in the form of alternating stationary states).

**Table 1:** **Estimation of model parameters value**.

	**Parameter**	**Value**	**Units**
α	Neurite growth rate	1.5–22	$\frac{\mu m}{\mu M\cdot h}$
The rate for GTP-tubulin association with microtubule is generally considered in the range of 10^6^−10^7^ *M*^−1^·*s*^−1^ (Mitchison and Kirschner, 1984; Desai and Mitchison, 1997). Using estimation of microtubule structural parameters (tubulin dimer size is 8 nm; microtubules are formed by the parallel association of 13 protofilaments, so 1 μm of microtubule consist 1625 tubulin dimers), neurite growth rate can be calculated: $10^{6}-10^{7}\frac{1}{M\cdot s}=\left(10^{6}-10^{7}\right)\cdot 10^{-6}\cdot \frac{3600}{1625}\frac{\mu m}{\mu M\cdot h}=2.2-22\frac{\mu m}{\mu M\cdot h}$ It should be noted that the estimation was performed in assumption than all microtubules grow simultaneously, but in practice their dynamics is highly desynchronized (some microtubules are shrinking, giving building material for neighbors' polymerization). Thus, the lower limit of neurite elongation rate should be decreased. In this paper the values in the range of 1.5–22 are considered.			
β	Neurite contraction rate	0.2–99	$\frac{\mu m}{h}$
According to experimental data of tubulin dissociation rate measurement it lies in the range 0.1 to 45 dimers s^−1^ (Walker et al., 1988; Drechsel et al., 1992; Desai and Mitchison, 1997). Using the algorithm described above the interval mentioned can be projected to 0.2–99 μM·h^−1^.			
*C*_0_	Tubulin concentration in the proximal part of neurite	5–20	μ*M*
Values are reported in (Gard and Kirschner, 1987; Walker et al., 1988).			
τ	The rate of building material (tubulin structures) degradation	0.0004–4	*h*^−1^
The range of acceptable values is determined by the individual microtubules half-life, which is 5–10 min. On the other hand, the upper bound is limited by the tubulin dimer degradation constant—several days (Sjöstrand and Karlsson, 1969; Hoffman and Lasek, 1975; Miller and Samuels, 1997).			
*V*_*at*_	The rate of active transport	12–100	$\frac{\mu m}{hour}$
Values are reported in (Lasek et al., 1984; Brown, 2000).			

To describe the evolution of the tubulin concentration along the neurite, the following approach was used. Let's consider number of tubulin structures moving along toward growth cone at constant velocity *V*_*at*_. There is also the degradation of the building material (e.g., oligomers or fragments of microtubules) during their transport. Consequently, the number of structures can be formulated in the following form:

(1) $$\begin{array}{l} \{\begin{array}{c} \frac{dN(x)}{dt}=-\tau N(x)\\ \frac{dx}{dt}=V_{at} \end{array}\Rightarrow dN\left(x\right)=-\frac{\tau N\left(x\right)}{V_{at}}dx\\ \;\Rightarrow N\left(x\right)=N_{0}e^{-\frac{\tau x}{V_{at}}} \end{array}$$

Where, *N*_0_ is the number of structures localized in the proximal part of the neurite (near the cell soma), *N*(*x*)—at the distance *x* along the neurite (*x* ∈ [0, *L*], *L* is a total length of neurite), *V*_*at*_ is the speed of the active transport, and τ is a constant describing the rate of building material (tubulin structures) degradation. The resulting equation can be expressed in terms of concentration. Thus a concentration profile in case of shape independent model (the cross-sectional area is constant along the neurite) can be represented as

(2) $$\begin{array}{r} C\left(x\right)=C_{0}e^{-\frac{\tau x}{V_{at}}} \end{array}$$

Where, *C*_0_ denotes the tubulin concentration in the proximal part of the neurite (its value is equal to concentration of building proteins in the cell soma) and *C*(*x*) is the profile of the tubulin concentration along the neuronal outgrowth. The concentration of the building material localized in a growth cone (*C*_*gc*_) available for neurite elongation can be obtained from Equation (2) as *C*_*gc*_ = *C*(*L*) (where *L* is a total length of the neurite). Assuming that the intensity of microtubule polymerization is a function of available building protein, the rate of the elongation process in a quasi-steady-state approximation can be expressed in the following form:

(3) $$\begin{array}{r} \frac{dL}{dt}=\alpha C_{gc}-\beta =\alpha C_{0}e^{-\frac{\tau L}{V_{at}}}-\beta \end{array}$$

Where, α and β are constants representing rates of neurite growth and contraction, respectively.

It follows from Equation (3) that the elongation rate decreases exponentially with increasing neurite length. Moreover, when the concentration in the growth cone reaches some critical level (*C*_*gc*_ = *C*_*cr*_ = *b/a*) the neurite growth stops (microtubule assembly is fully compensated by depolymerization process). Thus, the maximum neurite length is defined by the following value

(4) $$\begin{array}{r} L^{*}=\frac{V_{at}}{\tau }ln\left(\frac{C_{0}}{C_{cr}}\right) \end{array}$$

Note that the model accounts indirectly for mechanisms specific for dendrite and axon development. In particular, in defining the different values for the active transport velocity, one can take into account the influence of the microtubule orientation in dendrites and in axons (Heidemann et al., 1981; Baas et al., 1988) and the contribution of tubulin post-translational modification, which determines the spectrum of molecular motors that may participate in the cargo transport along microtubules (Reed et al., 2006; Bulinski, 2007; Konishi and Setou, 2009). Variations of the tubulin structure degradation rate reflect specific mechanisms of the axon and dendrite development associated with different proteins (maps/tau proteins) involved in the stabilization of the microtubules.

### Shape-dependent model

Next, we analyzed the effect of neurite geometry on the elongation dynamics. Our results suggested that the shape, e.g., neurite tapering with the distance from cell soma, may have a significant impact on the developmental dynamics. To account for this we made the following assumptions. First we assumed that the neurite cross-section near the growth cone is fixed and does not depend on the neurite length. Second, a possible source of soluble tubulin for neurite caliber enlargement may be a building material, which is released during depolymerization of microtubules transported. Thus, it does not reach the growth cone, while it can be used for the local changes in the neurite. Third, for simplicity, we assumed that the cross-section at any distance from the soma has a circular shape with a radius, *R*(*x, L*), which changes with the distance from the soma and total neurite length. Because cross-sectional area near the growth cone is assumed to be constant, the following boundary condition should be satisfied: *R*(*x, L*)|_*x* = *L*_ = *r*. Thus, using Equation (1), we obtained the following relationship:

(5) $$\begin{array}{rcl} C\left(x,L\right)S\left(x,L\right) & = & C\left(0,L\right)S{\left(0,L\right)e}^{-\frac{\tau x}{V_{at}}}\\ \Rightarrow C\left(x,L\right) & = & C_{0}\frac{S\left(0,L\right)}{S\left(x,L\right)}e^{-\frac{\tau x}{V_{at}}} \end{array}$$

Where *S*(*x, L*) is the cross-section area and *C*(*x, L*) is the concentration profile along the neurite (*x* ∈ [0, *L*]), *S*(*0, L*) denotes the cross-section area of the proximal part of the neuronal outgrowth. In this case, the concentration of building material localized in the growth cone (*x* = *L*) and available for neurite elongation is defined by the following Equation:

(6a) $$\begin{array}{r} CR=\{\begin{array}{cc} \frac{R\left(0,L\right)}{r}, & if\;\frac{R\left(0,L\right)}{r}<GR\\ GR=const, & otherwise \end{array} \end{array}$$

(6b) $$\begin{array}{r} C_{gc}=C_{0}\cdot CR^{2}\cdot e^{-\frac{\tau L}{V_{at}}}\Rightarrow \frac{dL}{dt}=\alpha C_{0}\cdot CR^{2}\cdot e^{-\frac{\tau L}{V_{at}}}-\beta \end{array}$$

where *R*(*0, L*) and *r* denote the radius of the cross-section near the cell soma and the growth cone, respectively. Also geometrical restriction (*GR*) introduced here meaning that proximal segment radius cannot exceed the soma size. Thus, tubulin concentration in the growth cone depends on the caliber ratio (*CR*) *R*(*0, L*)*/r*.

To illustrate how the neurite geometry influences the elongation dynamics, we investigated two options of *R*(*x, L*) dependence. First, we considered the linear dependence of the radius on the total length of the neurite, i.e., *R*(*x, L*) = *r* (*1 + k* (*L - x*)), *R*(0, *L*) = *r* (1+*k L*) where *k* is a tapering constant. It follows from Equation (6) that

(7) $$\begin{array}{r} C_{gc}=C_{0}{\left(1+kL\right)}^{2}e^{-\frac{\tau L}{V_{at}}}\Rightarrow \frac{dL}{dt}=\alpha C_{0}{\left(1+kL\right)}^{2}e^{-\frac{\tau L}{V_{at}}}-\beta \end{array}$$

**Figure 1:**
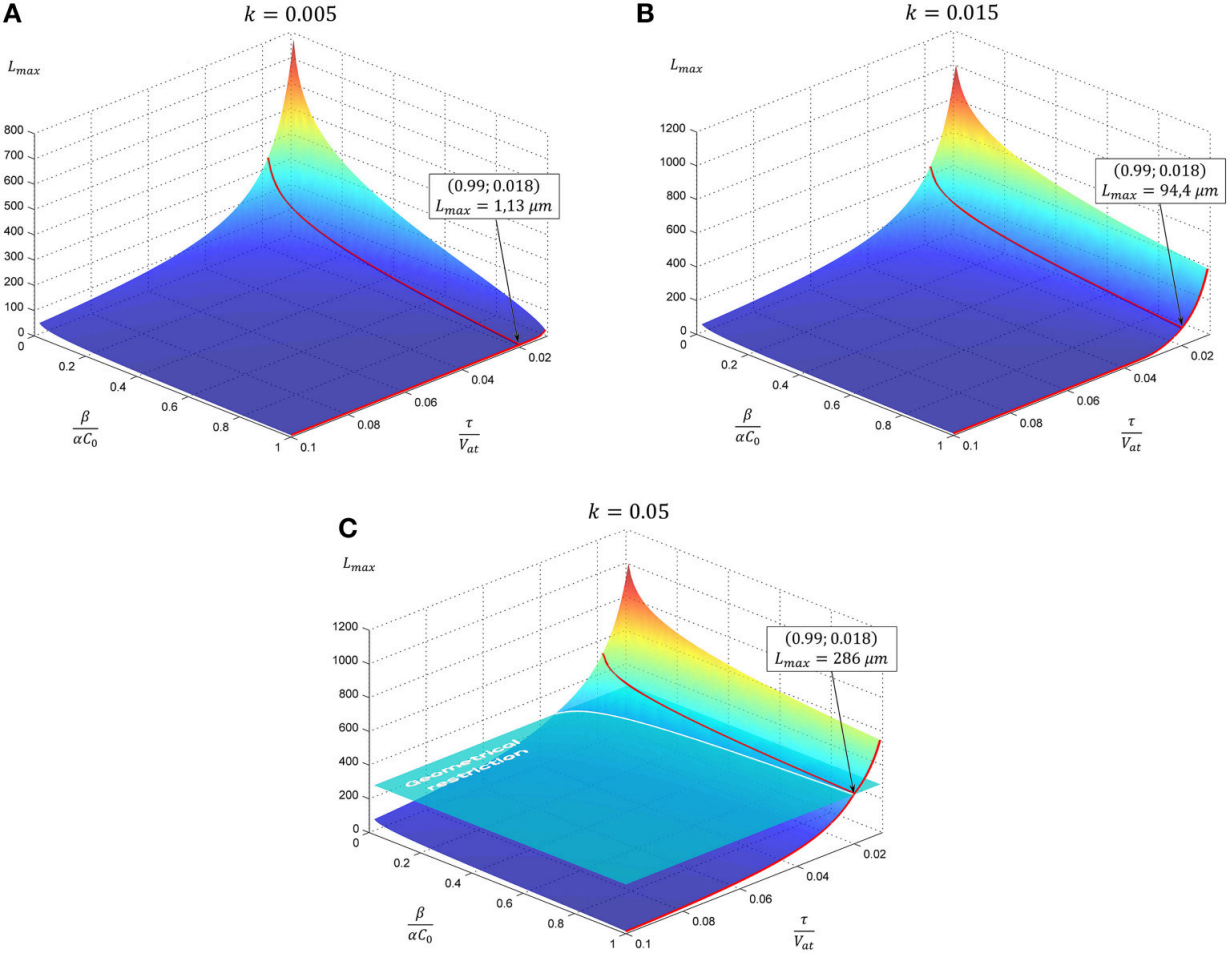
**The evolution of neurite maximal length depending on model parameters**. In subsequent panels results for the case of linear tapering are presented. The geometrical restriction (GR) represents the case when the proximal segment radius reaches the soma size. Parameter *k* characterizing neurite narrowing is varied: **(A)** *k* = 0.005, **(B)** *k* = 0.015, **(C)** *k* = 0.05. All calculations are performed with GR = 15. Point marked corresponds to the case considered in Figure 2.

**Figure 2:**
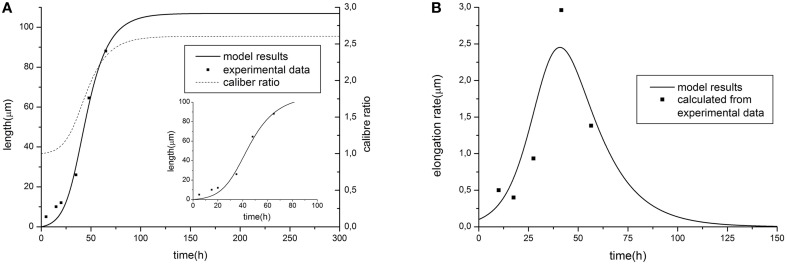
**Comparison of model predictions [(A) elongation dynamics and (B) rate for the case of linear tapering] with experimental data of dendrite growth (modified from (Teichmann and Shen, 2011), data represents the development of the DA9 motor neuron in wild-type *C. elegans*)**. Parameter values: α = 1.58, β = 9.38, *C*_0_ = 6, τ = 0.54, *V*_*at*_ = 30, *k* = 0.015. The value of caliber ratio represent the relationship between size of proximal and distal neurite part (R(0, L)/r) depending on time.

Resulting curves for maximal length and elongation dynamics depending on different parameters of the model are illustrated in Figure 1. Note that initially the elongation speed increases with the factor (*1* + *kL*)^2^, i.e., providing positive feedback. This is a cooperative effect of two processes. The first one is an increase of tubulin influx due to expansion of the proximal part. On the other hand, the increase of tubulin synthesis occurs, since, as previously noted, intracellular machinery maintains the tubulin concentration in soma at a fixed level. Thus, a feedback, stimulating the elongation process emerges. However, with increasing length, the portion of available tubulin decreases (building proteins need more time to travel from the cell soma to the growth cone, therefore, more tubulin structures degrade), hence slowing the elongation process. Note that this model prediction is in good agreement with experimental observations of dendrite growth (Dotti et al., 1988; Teichmann and Shen, 2011). Figure 2 illustrates the dendrite elongation dynamics of DA9 motor neuron in wild-type *C. elegans*. Analysis of the dendrite development of such type neuronal cells is interesting in the context of this paper, primarily because it forms single unbranched outgrowth. Thus, mentioned experimental model is very useful for verification of simulation results. It demonstrates the existence of a distinct phase of accelerated elongation that, in accordance with the proposed model can be explained by the presence of positive feedback, and the phase of slowing down growth, caused by the building protein degradation. Thus, it supports the prediction that the dendritic tapering with increasing distance to the cell soma may be linear or nearly linear.

Second, let us assume that the neurite radius transformation has the following form:

(8) $$\begin{array}{r} R\left(x,L\right)=re^{\frac{k\left(L-x\right)}{2}} \end{array}$$

i.e., the neurite cross-sectional area decreases exponentially with distance from the cell soma, and elongation dynamics can be described by the following expression:

(9) $$\begin{array}{r} \frac{dL}{dt}=\{\begin{array}{c} \alpha C_{0}e^{\left(k-\frac{\tau }{V_{at}}\right)L}-\beta ,\;if\;it\;it\;less\;than\;V_{at}\\ V_{at},\;otherwise \end{array} \end{array}$$

Note that the rate of elongation is limited by the geometric restriction, GR, and by the limit of active transport velocity, *V*_*at*_. Since neurite elongation requires tubulin, the overall growth rate is determined by building protein transport to the growth cone.

**Figure 3:**
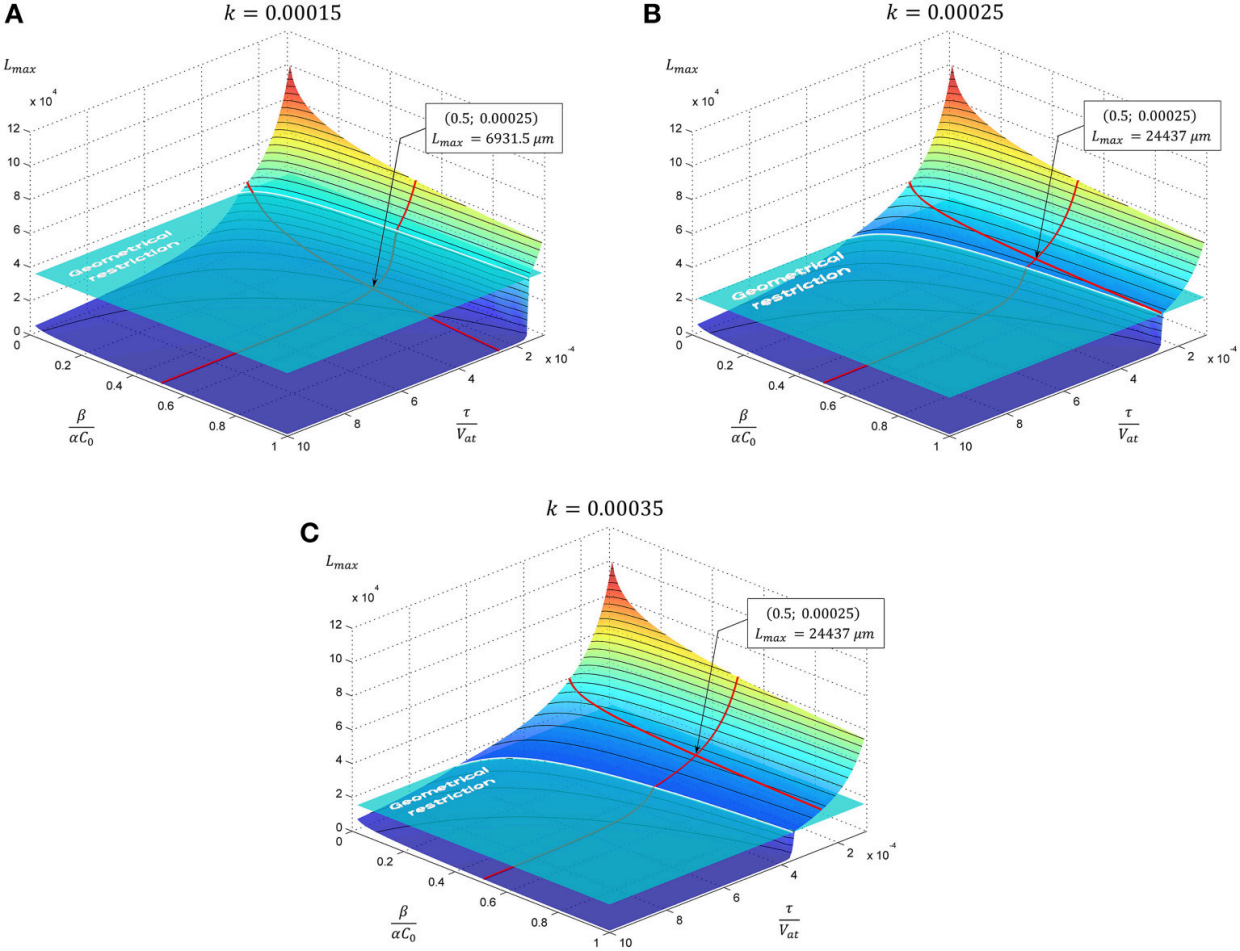
**The evolution of neurite maximal length depending on model parameters**. In subsequent panels results for the case of exponential decay of neurite cross-sectional area are presented. Tapering parameter *k* is varied: **(A)** *k* = 0.00015, **(B)** *k* = 0.00025, **(C)** *k* = 0.00035, geometrical restriction GR = 15. Point marked corresponds to the case considered in Figure 4.

**Figure 4:**
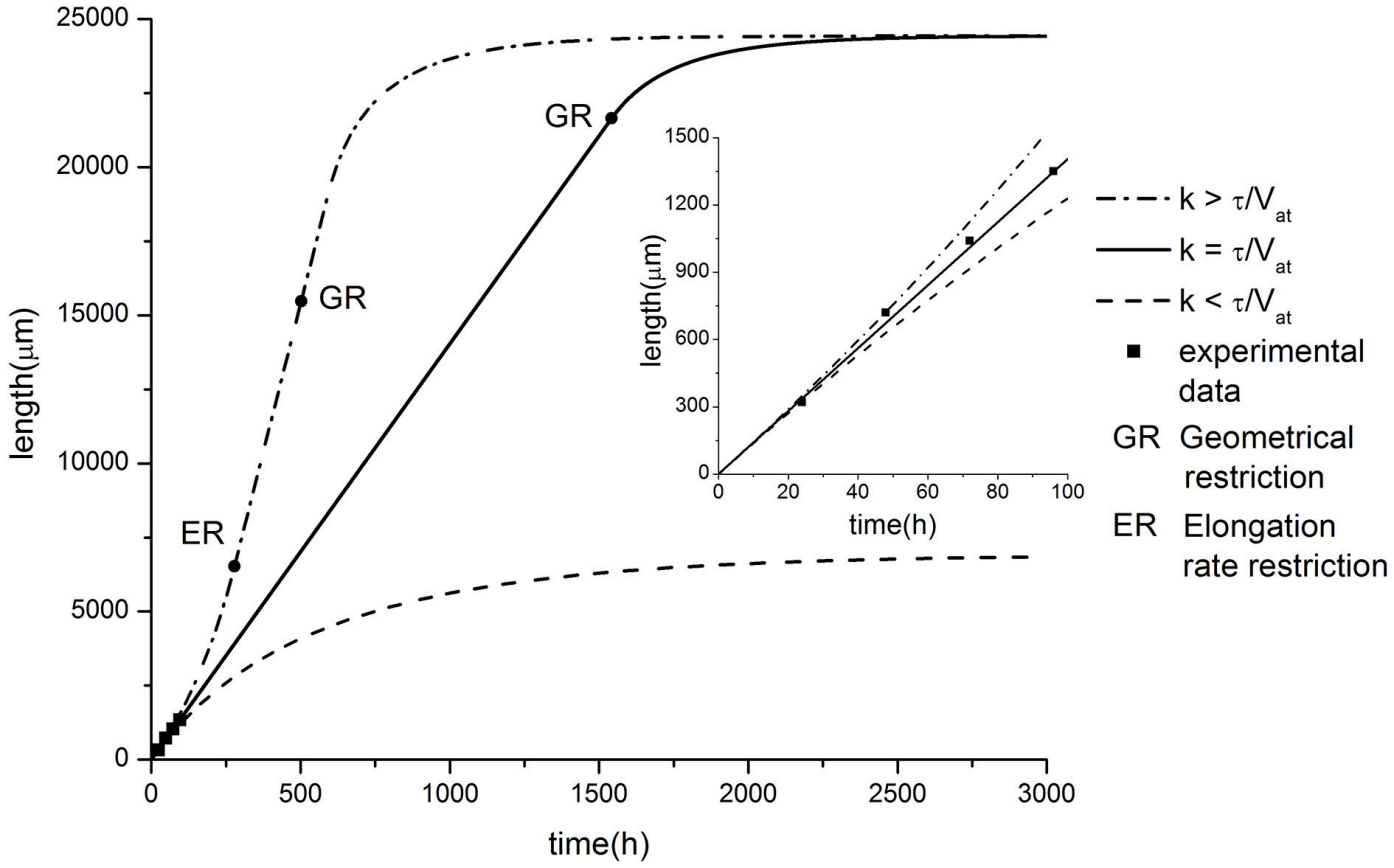
**Comparison of model predictions in case of exponential tapering with experimental data of axon growth (modified from Karten et al., 2005)**. Parameter values: α = 2.81, β = 14.06, *C*_0_ = 10, τ = 0.005, *V*_*at*_ = 40. Different elongation dynamics are illustrated for different values of parameter k: *k* = 0.00015 < τ/*V*_*at*_; *k* = 0.00025 = τ/*V*_*at*_; *k* = 0.00035 > τ/*V*_*at*_. The ER restriction means that the upper limit of neurite elongation rate determined by the active transport rate is achieved (dL/dt = *V*_*at*_).

The dependence of the neurite elongation dynamics on model parameters is illustrated in Figure 3. Interestingly, depending on the value *k* - τ*/V*_*at*_, one of three possible scenarios for the development processes can be realized (Figure 4).

Let us focus on the case of exact balance, i.e., *k* = τ/V_*at*_. As we noted earlier, typically, tubulin structures decay exponentially with increasing distance from the soma. However, in this case, the proximal segment increases exponentially during the elongation process. This effect provides the additional building protein influx from the cell soma (but, the tubulin concentration in the neurite proximal part *C*_0_ remains constant) sufficient to compensate for the loss of tubulin associated with degradation during the transport over longer distances. Note also that the neurite segment cross-sectional area and its volume decrease with distance at the same rate as the number of tubulin structures moving toward the growth cone. Therefore, the building protein concentration in the growth cone can be sustained at some constant level regardless of the distance from the cell soma. Thus, in the case where *k* = τ/V_*at*_, the model predicts a sustainable elongation process with a fixed elongation rate:

(9a) $$\begin{array}{r} \frac{dL}{dt}=\alpha C_{0}-\beta \end{array}$$

As a validation of the presented hypothesis the comparison of numerical calculation results and experimental studies of axonal outgrowth is performed. Figure 4 shows the growth dynamics of axons of retinal ganglion cells isolated from the rat CNS (Karten et al., 2005). The experimental data demonstrate a linear neurite elongation throughout the experiment (Dotti et al., 1988; Rochlin et al., 1996; Karten et al., 2005) indicating that the building material flow remains unchanged. Nevertheless, the model predicts that the outgrowth length is again limited even in the balanced condition. In contrast to previous cases, we obtain geometrical restriction because the proximal segment radius cannot exceed the soma size. As a result, the building proteins inflow is saturated, since the further expansion of the neurite proximal part is impossible. Thus, the positive feedback which supports the neurite elongation disappears and finally the growth stops.

When *k* < τ/V_*at*_, the neurite elongation dynamics were quite similar to the linear case considered earlier. The proximal segment expansion resulted in an increase in the amount of building proteins transported from the soma to the growth cone. However, this increase compensated for only a part of the tubulin loss due to degradation. As a result, the elongation process slows down over time and stops when a maximal length is reached (Figure 4).

For *k* > τ/V_*at*_, neurite growth dynamics occurred with increasing elongation speed. This effect can be explained as follows. The rate of the tubulin flow increase depends on the proximal segment size and in the case of *k* > τ/V_*at*_, it grows faster than losses occur due to the degradation of building proteins. This functions as positive feedback, accelerating the elongation process (Figure 4). However, similar to the case of constant speed elongation (*k* = τ/V_*at*_), the geometric constraint of limited proximal segment size provides a growth saturation factor.

Note that for this case there is a bias of the balance toward positive feedback, e.g., building proteins influx exceeds the amount of tubulin needed to compensate its degradation with increasing the neurite length. Thus, the outgrowth elongation accelerates with the course of time. However, the growth rate is limited by the active transport velocity and accelerated growth is replaced by the steady elongation.

## Discussion

We proposed a mathematical model of neurite development based on the microtubule cytoskeleton dynamics. The model incorporated basic molecular mechanisms underlying the elongation and demonstrated growth dynamics which is consistent with experimental studies of neural development (Dotti et al., 1988; Rochlin et al., 1996; Karten et al., 2005; Teichmann and Shen, 2011). In contrast to classical model approaches and existing experimental hypotheses considered differences in neurite structural organization (Baas et al., 1988; Sharp et al., 1997; Yu et al., 2000), selective post-translational microtubule modifications (Witte et al., 2008; Kollins et al., 2009) or accumulation of specific proteins (Takei et al., 2000; Gonzalez-Billault et al., 2001) as factors causing differences in the dynamics of dendrites and axons development (Bartlett and Banker, 1984) the main focus of the presented work is done on the effect of geometric changes occurring during the neurite elongation, and related scenarios of the outgrowth development.

Particularly, we found that the growth process can significantly depend on the neurite geometry. The geometrical transformation of the growth, e.g., changing the neurite radius with length, permitted us to delineate different types of the elongation dynamics. A dynamic mechanism of the geometry specific growth is concerned with interplay between factors promoting neurite growth and those suppressing it. Indeed, by the neurite elongation the proximal part expansion occurs and additional tubulin structures influx arise. At the same time, the tubulin synthesis increases since the intracellular machinery maintains its concentration at a fixed level in the cell soma. Thus, raising tubulin outflow to the neurite induced by geometrical changes and its consumption for elongation process is fully compensated by intracellular synthesis. As a result, the positive feedback promoting the neurite growth arises. On the other hand, the fraction of tubulin structures reaching growth cone decrease by the neurite elongation hence slows down the growth process. Moreover model implicitly accounts the consumption of the soluble tubulin released from degraded structures for the radial expansion (positive feedback formation).

Hence, depending on the parameters of the growth kinetics, several scenarios corresponding to dendrite or axon growth can be achieved. These types include the dendrite specific kinematics with slowing speed and axon growth specificity with constant elongation speed over long distances.

### Conflict of interest statement

The author declares that the research was conducted in the absence of any commercial or financial relationships that could be construed as a potential conflict of interest.
